# Association between asthma and COVID-19 severity during Omicron epidemic: a retrospective cohort study using real-world data

**DOI:** 10.1186/s12879-024-09520-9

**Published:** 2024-07-04

**Authors:** Huwen Wang, Xiaoting Jiang, Kate Ching Ching Chan, Yuchen Wei, Chi Tim Hung, Renee Wan Yi Chan, Conglu Li, Eman Yee Man Leung, Carrie Ho Kwan Yam, Tsz Yu Chow, Shi Zhao, Zihao Guo, Kehang Li, Ziqing Wang, Eng Kiong Yeoh, Ka Chun Chong

**Affiliations:** 1 School of Public Health and Primary Care, The Chinese University of Hong Kong, Hong Kong Special Administrative Region, China; 2 Department of Paediatrics, Prince of Wales Hospital, The Chinese University of Hong Kong, Hong Kong Special Administrative Region, China; 3 Centre for Health Systems and Policy Research, School of Public Health and Primary Care, The Chinese University of Hong Kong, Hong Kong Special Administrative Region, China

**Keywords:** Omicron, COVID-19 severity, Asthma, Inhaled corticosteroids

## Abstract

**Background:**

The available evidence presented inconsistencies and inconclusive findings regarding the associations between co-existing asthma and mortality among COVID-19 patients. The objective of the current study is to investigate the relationship between asthma and severe outcomes after SARS-CoV-2 Omicron infection in an infection-naïve population.

**Methods:**

A retrospective cohort study using propensity score matching was conducted. The COVID-19 patients requiring hospitalisation in Hong Kong from January 1, 2022, to November 13, 2022, an Omicron-predominated period, were identified. Severe clinical outcomes were defined as ICU admission and inpatient death after the first positive PCR results as well as a composite outcome of both.

**Results:**

Of the 74,396 hospitalised COVID-19 patients admitted, 1,290 asthma patients and 18,641 non-asthma patients were included in the matched cohort. The rates of death and the composite outcome were 15·3% and 17·2%, respectively, among the non-asthma patients,12·2% and 13·6%, respectively, among the asthma patients, with adjusted hazard ratios equal to 0·775 (95% CI: 0·660–0·909) and 0·770 (95% CI: 0·662–0·895), respectively. The negative association was more apparent in the elderly and female groups. Asthma remained a factor that lowered the risk of disease severity even though the patients were not fully vaccinated with at least two doses.

**Conclusions:**

We used real-world data to demonstrate that asthma was not a risk factor for COVID-19 severity of the infections of Omicron variant, even though the patients were not fully vaccinated.

**Supplementary Information:**

The online version contains supplementary material available at 10.1186/s12879-024-09520-9.

## Background

Asthma is the most prevalent chronic inflammatory lung disease. According to the Global Asthma Report 2018, asthma was estimated to affect over 300 million people worldwide [1]. Viral respiratory tract infections play a major role in asthma exacerbations, which is a leading cause of morbidity among asthma patients [2]. Since the beginning of the COVID-19 pandemic, concerns have been raised regarding the increased risk of asthma exacerbation and vulnerability to severe COVID-19 outcomes among asthmatics.

Several studies conducted in recent years have explored the relationship between pre-existing asthma and severe outcomes in COVID-19 patients. Generally, individuals with asthma and COVID-19 tend to face an elevated risk of intensive care unit (ICU) admission, hospitalization, and the need for ventilation [3]. However, the existing evidence has revealed inconsistencies and inconclusive results concerning the link between concurrent asthma and mortality in individuals with COVID-19 [3–6]. A substantial body of literature reported conflicting findings, with some studies suggesting that asthma increases the risk of mortality [7–10], while others showed no significant association [11–15] or even a decrease in risk [16–19] among COVID-19 patients.

﻿An inconsistency in the incidence of COVID-19 among asthmatics [5] and the association between asthma and COVID-19 in different settings make further investigation by region imperative. New evidence during the Omicron-predominated period is also lacking. Most importantly, few studies have used real-world data to evaluate the impact of asthma on the severe outcomes due to SARS-CoV-2 Omicron infection. Hence, in this study, we employed the official linked data including all registered infected cases, vaccination records, hospitalisation information, and death records to examine the relationship between asthma and the COVID-19 severity among hospitalised COVID-19 patients in Hong Kong.

## Methods

### Study design

This is a retrospective cohort study using propensity score matching to examine the association between asthma and severe outcomes among hospitalised COVID-19 patients. The nearest neighbour method without replacement was used in the propensity score matching, with the matching ratio set at 1:15 and the caliper set at 0·2 [20]. Logistic regression models were used to estimate the propensity score. The standardised mean difference (SMD) was used to assess covariate balance between asthma and non-asthma groups, with SMD < 0·1 indicating adequate balance [21].

### Data source

All hospitalised COVID-19 patients in Hong Kong admitted from January 1, 2022, to November 13, 2022 (study period), an Omicron-predominated period, were identified with inpatient and historical medication records retrieved from the Hong Kong Hospital Authority, a statutory body to manage all the 41 public hospitals in Hong Kong. During the pandemic, ﻿all COVID-19 hospitalisations were managed in the public hospital system in Hong Kong. The data were linked to the epidemiological investigation database and the COVID-19 vaccination registry database held by the Hong Kong Department of Health using unique pseudo key numbers to obtain extra information including medical history and vaccination status.

### Study population

Patients who were aged ≥ 18 years at hospital admission, with confirmed positive reverse transcription-polymerase chain reaction (RT-PCR) results, and had medication records and clinical records indicating severity outcomes (intensive care unit (ICU) admission and inpatient death) were included in the cohort.

In the primary analysis, asthma patients were defined as those with at least one ICD-9 CM code of 493 in the inpatient records during the three years prior to the hospital admission for COVID-19. The outpatient data and epidemiological investigation data were cross-checked to confirm the presence of the diagnosis. In the exploratory analysis, the asthma patients were further classified based on their prescribed inhaled corticosteroids (ICS) doses into the following groups (1) no/unknown ICS; (2) low-dose ICS; (3) medium-dose ICS; and (4) high-dose ICS. The ICS dose levels were defined based on guidelines from the Global Initiative for Asthma [22]. In assigning individuals to low, medium, or high ICS dose levels, prescriptions for ICS in the year prior to the first positive PCR date and the highest dose the individual was prescribed were used [7]. Results using the most recent dose the individual was prescribed [8] were also provided in the sensitivity analysis. In addition, the effect of asthma on COVID outcomes among patients with different asthma therapy was examined. Asthma patients with no therapy targeting asthma, with ICS + long-acting β2-agonists (LABA) / short-acting β2-agonists (SABA), and with ICS + LABA/SABA + long-acting muscarinic antagonists (LAMA) / leukotriene receptor antagonists (LTRA) / Xanthines (equivalent to the highest steps in the asthma management stepwise approach [22] and can be used to define severe asthma [23]) were respectively compared with their matched non-asthma counterparts.

### Outcomes

Severe outcomes were defined as ICU admission and inpatient death after the first positive PCR results as well as a composite outcome of either ICU admission or inpatient death. For those discharged without experiencing these events, their event time was censored on the discharge date of their last hospitalisation during the study period. For those who did not experience the events and were not discharged yet, their event time was censored on November 27, 2022, 14 days after the end date of data extraction, to avoid bias from those who had not had adequate time to accrue an outcome [23]. The event time was calculated as the number of days from the first positive PCR date to the first occurrence of the specific events.

### Covariates

Covariates matched included age, sex, vaccination status, use of paxlovid and molnupiravir (which proved to be effective in reducing the mortality and hospitalisation rates in patients with COVID-19) [24, 25], use of other anti-COVID-19 treatments (i.e., dexamethasone, remdesivir, baricitinib, tocilizumab, and interferon beta-1b), medical history, and calendar week. The vaccination status of the individuals was grouped into 0, 1, 2, and ≥ 3 doses. Only those who had taken the respective vaccine doses 14 days before the first positive PCR date were regarded as vaccinated with the dose, considering the latency between vaccine uptake and full development of immune responses.

Medical history was identified using the ICD-9 CM codes, including hypertension (401·X-405·X), diabetes (250·X), coronary artery disease (410·X—414·X), congestive heart failure (398·91, 402·01, 402·11, 402·91, 404·01, 404·03, 404·11, 404·13, 404·91, 404·93, 425·4—425·9, 428·X), arrhythmia (426·0, 426·13, 426·7, 426·9, 426·10, 426·12, 427·0—427·4, 427·6—427·9, 785·0, 996·01, 996·04, V45·0, V53·3), chronic obstructive pulmonary disease (COPD; 496), malignancy (140·X—172·X, 174·X—208·X, 238·6), cerebrovascular disease (362·34, 430·X—438·X), peripheral vascular disease (093·0, 437·3, 440·X, 441·X, 443·1—443·9, 447·1, 557·1, 557·9, V43·4), chronic liver disease (070·22, 070·23, 070·32, 070·33, 070·44, 070·54, 070·6, 070·9, 456·0—456·2, 570·X, 571·X, 572·2- 572·8, 573·3, 573·4, 573·8, 573·9, V42·7), chronic kidney disease (585·X), and obesity (278·0). The outpatient data and epidemiological investigation data were cross-checked to supplement the inpatient records of the medical history.

### Statistical analysis

Descriptive statistics are presented for patients with and without asthma. Cox proportional hazard models with weights and clusters representing the matching effect were conducted to examine the association between asthma and severe outcomes. All asthma patients, asthma patients prescribed with each level of ICS, and asthma patients with different therapy targeting asthma were compared respectively with their matched controls. Crude and adjusted hazard ratios (HR) that adjusted for sex, age, vaccination status, use of paxlovid, molnupiravir and other anti-COVID-19 treatments, medical history, and calendar week were respectively estimated and presented with their 95% confidence intervals (CIs). The distributions of the time-to-events among asthma and non-asthma patients were visually presented using the adjusted survival curves.

Subgroup analyses were conducted by age (< 65 years and ≥ 65 years), sex, and vaccination status. Sensitivity analyses included: 1) excluding those with COPD diagnoses from the cohort, 2) matching at a ratio of 1:1 and 1:2, and 3) using the most recent ICS doses to classify patients. A *p*-value < 0·05 was declared as a statistical significance. All analyses were conducted in R statistical software (version 4·1·1) (R Program for Statistical Computing) [26].

## Results

**Fig. 1:**
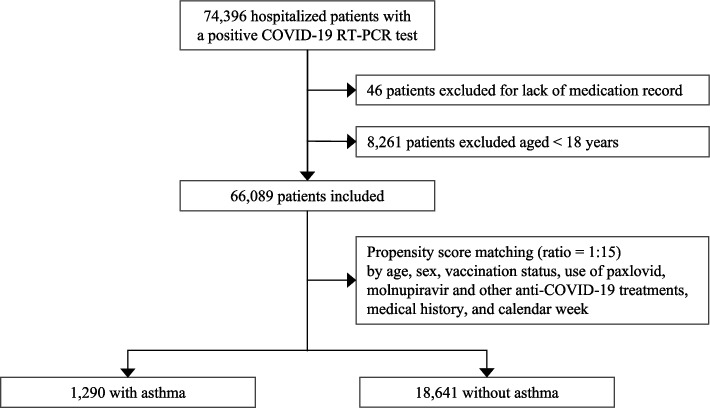
The procedure of including and matching study participants

Of the 74,396 hospitalised COVID-19 patients admitted from January 1, 2022, to November 13, 2022, 66,089 were included for matching, of which 1,290 (2·0%) were diagnosed with asthma (Fig. 1). Propensity score matching yielded adequate balance with SMDs for all covariates < 0·1 (eFigure 1). Patients’ characteristics after matching are shown in Table 1. Approximately half of the patients were ≥ 80 years old (non-asthma group: 47·8%, asthma group: 47·7%) and most were not fully vaccinated (vaccination dose < 2, non-asthma group: 51·7%, asthma group: 52·3%). Hypertension was the most frequent chronic condition in both groups.

**Table 1:** Characteristics of COVID-19 patients with and without asthma after matching

Characteristics	Without asthma (*n* = 18,641)	With asthma (*n* = 1,290)
Age, years (mean ± standard deviation)	74 ± 18	75 ± 18
Age group, n (%)		
18–49	2084 (11·2)	138 (10·7)
50–64	2134 (11·4)	148 (11·5)
65–79	5512 (29·6)	389 (30·2)
≥ 80	8911 (47·8)	615 (47·7)
Sex, n (%)		
Female	10827 (58·1)	741 (57·4)
Male	7814 (41·9)	549 (42·6)
Vaccination status, n (%)		
0 dose	7131 (38·3)	503 (39·0)
1 dose	2497 (13·4)	172 (13·3)
2 doses	4621 (24·8)	314 (24·3)
≥ 3 doses	4392 (23·6)	301 (23·3)
Use of paxlovid, n (%)	3758 (20·2)	257 (19·9)
Use of molnupiravir, n (%)	3839 (20·6)	264 (20·5)
Use of other anti-COVID-19 treatments^a^, n (%)	7359 (39·5)	526 (40·8)
Medical history, n (%)		
Hypertension	10,540 (56·5)	730 (56·6)
Diabetes	4895 (26·3)	329 (25·5)
Coronary artery disease	2916 (15·6)	207 (16·0)
Congestive heart failure	2871 (15·4)	210 (16·3)
Cerebrovascular disease	2846 (15·3)	193 (15·0)
Arrhythmia	2787 (15·0)	194 (15·0)
Chronic obstructive pulmonary disease	2434 (13·1)	208 (16·1)
Malignancy	1398 (7·5)	98 (7·6)
Obesity	1408 (7·6)	92 (7·1)
Chronic liver disease	1346 (7·2)	94 (7·3)
Chronic kidney disease	1314 (7·0)	86 (6·7)
Peripheral vascular disease	221 (1·2)	15 (1·2)

^a^Other anti-COVID-19 treatments include dexamethasone, remdesivir, baricitinib, tocilizumab, and interferon beta-1b

The risk of inpatient death and ICU admission or death was significantly lower among patients with asthma (Table 2 and eFigure 2). The rates of death and the composite outcome were 15·3% and 17·2% among the non-asthma patients, while 12·2% and 13·6% among the asthma patients, with adjusted hazard ratios (HRs) of death equal to 0·775 (95% confidence interval [CI]: 0·660–0·909) and ICU or death equal to 0·770 (95% CI: 0·662–0·895), respectively. This lower risk was also detected for ICU admission alone but not reaching statistical significance.

**Table 2:** Association between asthma and severe COVID-19 outcomes

	Without asthma n (%)	With asthma n (%)	Crude HR (95% CI)	*p*-value	Adjusted HR (95% CI)^a^	*p*-value
ICU	454 (2·4)	28 (2·2)	0·895 (0·611, 1·310)	0·568	0·897 (0·613, 1·312)	0·576
Death	2847 (15·3)	158 (12·2)	0·785 (0·670, 0·919)	**0·003**	0·775 (0·660, 0·909)	**0·002**
ICU or death	3213 (17·2)	176 (13·6)	0·768 (0·661, 0·892)	**< 0·001**	0·770 (0·662, 0·895)	**< 0·001**

*HR* Hazard ratio, *CI* Confidence interval

^a^Adjusted for age, sex, vaccination status, use of paxlovid, molnupiravir and other anti-COVID-19 treatments, medical history, and calendar week

When grouping asthma patients based on their prescribed ICS dose levels or asthma therapy, the results for inpatient death and the composite outcome were generally consistent with the primary analysis (Table 3 and eTable 1). The protective effect of asthma on death and the composite outcome was the most apparent among asthma patients who were not prescribed ICS (adjusted HR (95% CI): death: 0·669 (0·459, 0·977); ICU or death: 0·675 (0·480, 0·950)) and who received no therapy targeting asthma (adjusted HR (95% CI): death: 0·237 (0·031, 1·834); ICU or death: 0·375 (0·091, 1·546)). The effect was not as strong among severe asthma patients (i.e., patients prescribed high-dose ICS or ICS + LABA/SABA + LAMA/LTRA/ Xanthines) but remained protective. The adjusted HRs (95% CI) among patients prescribed high-dose ICS and patients prescribed ICS + LABA/SABA + LAMA/LTRA/Xanthines were 0·836 (0·573, 1·219) and 0·819 (0·641, 1·046) respectively for death, and 0·916 (0·649, 1·292) and 0·837 (0·663, 1·056) respectively for the composite outcome.

**Table 3:** Association between asthma and severe COVID-19 outcomes stratified by the highest ICS dose in the year before the first positive PCR date

	Without asthma n (%)	With asthma n (%)	Crude HR (95% CI)	*p*-value	Adjusted HR (95% CI)^a^	*p*-value
**Patients with asthma not prescribed ICS (*n* = 339) vs. patients without asthma (*n* = 5,026)**						
ICU	121 (2·4)	9 (2·7)	1·095 (0·553, 2·168)	0·794	1·047 (0·528, 2·077)	0·895
Death	743 (14·8)	29 (8·6)	0·572 (0·397, 0·824)	**0·003**	0·669 (0·459, 0·977)	**0·037**
ICU or death	842 (16·8)	34 (10·0)	0·580 (0·415, 0·812)	**0·002**	0·675 (0·480, 0·950)	**0·024**
**Patients with asthma prescribed low-dose ICS (*n* = 259) vs. patients without asthma (*n* = 3,766)**						
ICU	89 (2·4)	3 (1·2)	0·508 (0·159, 1·630)	0·255	0·558 (0·173, 1·798)	0·328
Death	582 (15·5)	31 (12·0)	0·859 (0·597, 1·237)	0·414	0·978 (0·688, 1·392)	0·903
ICU or death	647 (17·2)	32 (12·4)	0·767 (0·537, 1·096)	0·145	0·866 (0·615, 1·219)	0·409
**Patients with asthma prescribed medium-dose ICS (*n* = 477) vs. patients without asthma (*n* = 6,779)**						
ICU	165 (2·4)	9 (1·9)	0·758 (0·388, 1·483)	0·418	0·766 (0·394, 1·490)	0·433
Death	1049 (15·5)	67 (14·0)	0·831 (0·656, 1·051)	0·123	0·739 (0·580, 0·941)	**0·014**
ICU or death	1185 (17·5)	73 (15·3)	0·801 (0·638, 1·007)	0·057	0·731 (0·579, 0·923)	**0·008**
**Patients with asthma prescribed high-dose ICS (*n* = 215) vs. patients without asthma (*n* = 3,070)**						
ICU	79 (2·6)	7 (3·3)	1·314 (0·620, 2·783)	0·476	1·361 (0·630, 2·940)	0·433
Death	473 (15·4)	31 (14·4)	0·906 (0·623, 1·317)	0·605	0·836 (0·573, 1·219)	0·352
ICU or death	539 (17·6)	37 (17·2)	0·967 (0·684, 1·367)	0·849	0·916 (0·649, 1·292)	0·617

*HR* Hazard ratio, *CI* Confidence interval, *ICS *Inhaled corticosteroids

^a^Adjusted for age, sex, vaccination status, use of paxlovid, molnupiravir and other anti-COVID-19 treatments, medical history, and calendar week

In the subgroup analysis by age and sex, the protective effect of asthma on inpatient death and the composite outcome was more apparent in the elderly groups and females compared with the young age groups and males respectively (Table 4). The presence of asthma still lowered the risk of disease severity even in the patients who were not fully vaccinated (i.e., vaccinated with 0 or 1 dose). The analyses after excluding those with COPD, by setting the matching ratio at 1:1 and 1:2, or using the most recent ICS dose to classify patients, yielded results similar to the primary analyses (eFigure 3–5 and eTable 2–4).

**Table 4:** Subgroup analysis of the association between asthma and severe COVID-19 outcomes stratified by age, sex, and vaccination status

	Without asthma n (%)	With asthma n (%)	Crude HR (95% CI)	*p*-value	Adjusted HR (95% CI)	*p*-value
**Stratified by age**^**a**^						
Aged < 65 years: asthma (*n* = 286) vs. no asthma (*n* = 4,218)						
ICU	137 (3·2)	9 (3·1)	0·993 (0·506, 1·949)	0·984	1·036 (0·529, 2·029)	0·919
Death	150 (3·6)	8 (2·8)	0·932 (0·454, 1·913)	0·848	1·102 (0·538, 2·257)	0·791
ICU or death	271 (6·4)	14 (4·9)	0·812 (0·473, 1·394)	0·451	0·897 (0·520, 1·547)	0·696
Aged ≥ 65 years: asthma (*n* = 1,004) vs. no asthma (*n* = 14,423)						
ICU	317 (2·2)	19 (1·9)	0·865 (0·545, 1·373)	0·538	0·875 (0·551, 1·390)	0·572
Death	2697 (18·7)	150 (14·9)	0·766 (0·652, 0·900)	**0·001**	0·751 (0·636, 0·886)	**< 0·001**
ICU or death	2942 (20·4)	162 (16·1)	0·758 (0·649, 0·887)	**< 0·001**	0·757 (0·646, 0·887)	**< 0·001**
**Stratified by sex**^**b**^						
Male: asthma (*n* = 549) vs. no asthma (*n* = 7,814)						
ICU	230 (2·9)	14 (2·6)	0·888 (0·518, 1·520)	0·664	0·823 (0·480, 1·409)	0·477
Death	1427 (18·3)	84 (15·3)	0·889 (0·720, 1·098)	0·276	0·868 (0·695, 1·083)	0·210
ICU or death	1601 (20·5)	92 (16·8)	0·857 (0·700, 1·050)	0·136	0·856 (0·696, 1·054)	0·143
Female: asthma (*n* = 741) vs. no asthma (*n* = 10,827)						
ICU	224 (2·1)	14 (1·9)	0·918 (0·535, 1·576)	0·757	0·973 (0·566, 1·674)	0·923
Death	1420 (13·1)	74 (10·0)	0·701 (0·554, 0·887)	**0·003**	0·683 (0·540, 0·865)	**0·002**
ICU or death	1612 (14·9)	84 (11·3)	0·699 (0·560, 0·873)	**0·002**	0·689 (0·552, 0·859)	**< 0·001**
**Stratified by vaccination status**^**c**^						
0 or 1 dose: asthma (*n* = 675) vs. no asthma (*n* = 9,628)						
ICU	217 (2·3)	18 (2·7)	1·188 (0·736, 1·916)	0·481	1·213 (0·750, 1·960)	0·431
Death	2298 (23·9)	124 (18·4)	0·731 (0·612, 0·874)	**0·001**	0·749 (0·625, 0·899)	**0·002**
ICU or death	2453 (25·5)	136 (20·1)	0·754 (0·635, 0·896)	**0·001**	0·781 (0·656, 0·930)	**0·006**
2 or 3 doses: asthma (*n* = 615) vs. no asthma (*n* = 9,013)						
ICU	237 (2·6)	10 (1·6)	0·624 (0·330, 1·180)	0·147	0·597 (0·316, 1·128)	0·112
Death	549 (6·1)	34 (5·5)	0·985 (0·705, 1·377)	0·930	0·914 (0·659, 1·268)	0·592
ICU or death	760 (8·4)	40 (6·5)	0·796 (0·584, 1·085)	0·149	0·741 (0·547, 1·003)	0·052

*HR* Hazard ratio, *CI* Confidence interval

^a^The adjusted HR was estimated after adjusting for sex, vaccination status, use of paxlovid, molnupiravir and other anti-COVID-19 treatments, medical history, and calendar week

^b^The adjusted HR was estimated after adjusting for age, vaccination status, use of paxlovid, molnupiravir and other anti-COVID-19 treatments, medical history, and calendar week

^c^The adjusted HR was estimated after adjusting for age, sex, use of paxlovid, molnupiravir and other anti-COVID-19 treatments, medical history, and calendar week

## Discussion

Conflicting findings of COVID-19 severity in individuals with asthma were reported in literature. In this study, we showed that asthma was associated with a lower risk of the severe COVID-19 outcomes, consistent with a retrospective population study demonstrating that patients with allergic asthma had a significantly lower hospitalisation for COVID-19 [27]. Several mechanisms can explain the relationship. Firstly, low gene expression of the SARS-CoV-2 viral entry receptor angiotensin-converting-enzyme-2 (ACE2) is associated with atopic asthma, limiting the entry of coronavirus into the cell, thus leading to lesser COVID-19 severity [28]. The mechanism is supported by a study examining the differential expression of ACE2 in upper and lower airway cells of three cohorts of children and adults with respiratory allergy and asthma [29]. In addition, patients with allergic asthma also develop type 2 immune responses, resulting in an increased production of cytokines such as interleukin-13, which significantly decreases ACE2 gene expression [28]. Moreover, interleukin-13 could reduce intracellular viral load and cell-to-cell transmission, thus limiting the virus’s ability to relocate to deeper airways to trigger more severe disease [30]. Interlekin-13 is also known to upregulate MUC5AC, a major airway mucin involved in asthma, making patients with allergic asthma less susceptible to severe COVID-19. Secondly, mucus hypersecretion shield SARS-CoV-2 from reaching the type 2 alveolar cells in the distal lung epithelium, where ACE2 is predominantly expressed [31].

Our study found that the protective effect of asthma was more pronounced in the elderly and females. In general, females had less severe COVID-19 outcomes when compared to males, regardless of the effect of asthma. It could be due to sex differences in certain physiological variables such as pro-inflammatory cytokines and T cell activation [32]. In addition, studies have suggested that severe asthma is associated with older age due to a decline in lung function such as a two-fold reduction in FEV_1_%, and that females experience a later onset of asthma with more severe symptoms, possibly due to the role of female sex hormones [33]. The sex-difference association could also be explained by females having more sputum eosinophils and fewer ACE2 receptors than males, suggesting males had a higher expression of ACE2-associated genes in bronchial biopsy and bronchoalveolar lavage [34].

Currently, the impact of asthma severity on COVID-19 outcomes remains unclear. In our study, patients with asthma prescribed with lowering medicine-dose ICS had better COVID-19 outcomes compared with patients without asthma, whereas patients with asthma prescribed with high-dose ICS did not, though we acknowledged that the sample size in the subgroup analysis was small. Several large observational studies [7, 8, 23] stratified asthma severity by prescribed medications (e.g., patients prescribed lower dose ICS were defined as mild patients, while those prescribed higher dose ICS as more severe patients [7]) and found a worse outcome in patients with severe asthma. Based on existing evidence, we speculate that better control of asthma may account for the differences in the risk of COVID-19 severity.

The major strength of our study is that we utilized data from a predominantly Omicron variant period, whereas most studies [7, 8, 10, 23] conducted their analysis prior to the Omicron outbreak when infected cases typically presented with more severe clinical manifestations [35]. In addition, none of these studies have adjusted for the necessary pharmaceutical interventions. In contrast, our study has controlled for the effects of both COVID-19 vaccination and antivirals in order to minimize their influence on the relationship between asthma and COVID-19 severity. Also, few investigations [23] have specifically focused on studying hospitalized patients with COVID-19, who typically present moderate-to-severe symptoms. Our study population is restricted to hospitalized patients, which enhances the relevance to this critical patient population. Additionally, the inpatient data were obtained from all public hospitals in Hong Kong, where approximately 90% of hospitalization services are provided by the public sector. Therefore, our real-world data ensures representativeness at the territory level.

Our study has several limitations. First, hospitalisations with COVID-19 infection cannot be differentiated from hospitalisation due to COVID-19 infection while using health administrative data. Second, asthma was defined based on diagnostic codes in the current study and not from the original medical diagnosis through medical history and objective test results. ﻿Mild asthma patients with intermittent symptoms might have been excluded, which may decrease the generalizability of the findings. Third, asthma severity was stratified by prescribed asthma medications. Compliance with the prescription could not be obtained using the medical records, which may incur potential bias. Fourth, included patients were mainly infected with the Omicron variants. Therefore, the findings may not be generalised to other genetic variants of SARS-CoV-2. Fifth, as with many retrospective cohort studies employing a registry database, only limited information on covariates was available in this study. Confounders such as smoking history and body mass index were not included in the database and thus were not controlled in the analysis. Sixth, the definition of severe clinical outcomes varies across studies. The current study used ICU admission and inpatient death as a proxy for severe COVID-19 outcomes. Other outcomes including mechanical ventilation were not included due to the limit of data availability. Lastly, the results of ICU admission should be interpreted with caution because ICU capacity was overwhelmed by the surge of cases during the study period.

## Conclusion

We used real-world data to demonstrate that asthma was not a risk factor for severe COVID-19 outcomes, including ICU admission and death. The protective effect of asthma on inpatient death and the composite outcome was even more apparent in the elderly and female groups as well as the less severe asthma patients. It’s imperative for asthma patients to well control their asthma in the context of COVID-19 pandemic. Our higher-quality data improve the understanding about the role of asthma in COVID-19 severity, especially due to the infections of Omicron variant.

### Supplementary Information

Supplementary Material 1.

## Data Availability

The data that support the findings of this study are available from the Hospital Authority and Department of Health, Hong Kong Government but restrictions apply to the availability of these data, which were used under license for the current study, and so are not publicly available. Data are however available from the corresponding author (KCC) upon reasonable request and with permission of the Hospital Authority and Department of Health, Hong Kong Government.

## References

[1] The Global Asthma Report. Auckland: Global Asthma Network; 2018. http://globalasthmareport.org/2018/resources/Global_Asthma_Report_2018.pdf. Accessed 20 Nov 2023.

[2] Busse WW, Lemanske RF, Gern JE (2010). Role of viral respiratory infections in asthma and asthma exacerbations. The Lancet.

[3] US Centers for Disease Control and Prevention. Brief Summary of the Association between Underlying Conditions and Severe COVID-19: Asthma. https://www.cdc.gov/coronavirus/2019-ncov/science/science-briefs/pdf/K-Brief-Summary-of-Findings-on-the-Association-Between-Asthma-and-SevereCOVID-19-Outcomes-508.pdf. Accessed 20 Nov 2023.

[4] Adir Y, Saliba W, Beurnier A, Humbert M (2021). Asthma and COVID-19: an update. Eur Respir Rev.

[5] Skevaki C, Karsonova A, Karaulov A, Xie M, Renz H (2020). Asthma-associated risk for COVID-19 development. J Allergy Clin Immunol.

[6] Sunjaya AP, Allida SM, Di Tanna GL, Jenkins CR (2022). Asthma and COVID-19 risk: a systematic review and meta-analysis. Eur Respir J.

[7] Dolby T, Nafilyan V, Morgan A, Kallis C, Sheikh A, Quint JK. Relationship between asthma and severe COVID-19: a national cohort study. Thorax. 2022;78(2):120-27. 10.1136/thoraxjnl-2021-218629 PMC8983409 35354646

[8] Schultze A, Walker AJ, MacKenna B (2020). Risk of COVID-19-related death among patients with chronic obstructive pulmonary disease or asthma prescribed inhaled corticosteroids: an observational cohort study using the OpenSAFELY platform. Lancet Respir Med.

[9] Akhtar H, Khalid S, Rahman Fu (2021). Presenting Characteristics, Comorbidities, and Outcomes Among Patients With COVID-19 Hospitalized in Pakistan: Retrospective Observational Study. JMIR Public Health Surveill..

[10] Yong Jun C, Ju-Young P, Hye Sun L (2021). Effect of asthma and asthma medication on the prognosis of patients with COVID-19. Eur Respir J.

[11] Aveyard P, Gao M, Lindson N (2021). Association between pre-existing respiratory disease and its treatment, and severe COVID-19: a population cohort study. Lancet Respir Med.

[12] Castilla J, Guevara M, Miqueleiz A (2021). Risk factors of infection, hospitalization and death from SARS-CoV-2: a population-based cohort study. J Clin Med.

[13] Hansen ESH, Moeller AL, Vibeke B (2021). Severe outcomes of COVID-19 among patients with COPD and asthma. ERJ Open Research.

[14] Ren J, Pang W, Luo Y (2022). Impact of allergic rhinitis and asthma on COVID-19 infection, hospitalization, and mortality. J Allergy Clin Immunol Pract..

[15] Parra-Bracamonte GM, Lopez-Villalobos N, Parra-Bracamonte FE (2020). Clinical characteristics and risk factors for mortality of patients with COVID-19 in a large data set from Mexico. Ann Epidemiol.

[16] Yang JM, Koh HY, Moon SY (2020). Allergic disorders and susceptibility to and severity of COVID-19: a nationwide cohort study. J Allergy Clin Immunol.

[17] Guillaume B, Jonathan C, Anne-Sophie M (2021). Chronic respiratory diseases are predictors of severe outcome in COVID-19 hospitalised patients: a nationwide study. Eur Respir J.

[18] Calmes D, Graff S, Maes N (2021). Asthma and COPD Are Not Risk Factors for ICU Stay and Death in Case of SARS-CoV2 Infection. J Allergy Clin Immunol Pract..

[19] Sousa BL, Brentani A, Ribeiro CC (2021). Non-communicable diseases, sociodemographic vulnerability and the risk of mortality in hospitalised children and adolescents with COVID-19 in Brazil: a cross-sectional observational study. BMJ Open.

[20] Austin PC (2011). Optimal caliper widths for propensity-score matching when estimating differences in means and differences in proportions in observational studies. Pharm Stat.

[21] Austin PC (2009). Using the standardized difference to compare the prevalence of a binary variable between two groups in observational research. Commun Stat Simul Comput.

[22] Global Initiative for Asthma. Global Strategy for Asthma Management and Prevention. 2022. www.ginasthma.org.

[23] Bloom CI, Drake TM, Docherty AB (2021). Risk of adverse outcomes in patients with underlying respiratory conditions admitted to hospital with COVID-19: a national, multicentre prospective cohort study using the ISARIC WHO Clinical Characterisation Protocol UK. Lancet Respir Med.

[24] Wong CKH, Au ICH, Lau KTK, Lau EHY, Cowling BJ, Leung GM (2022). Real-world effectiveness of early molnupiravir or nirmatrelvir-ritonavir in hospitalised patients with COVID-19 without supplemental oxygen requirement on admission during Hong Kong's omicron BA.2 wave: a retrospective cohort study. Lancet Infect Dis.

[25] Wong CKH, Au ICH, Lau KTK, Lau EHY, Cowling BJ, Leung GM (2022). Real-world effectiveness of molnupiravir and nirmatrelvir plus ritonavir against mortality, hospitalisation, and in-hospital outcomes among community-dwelling, ambulatory patients with confirmed SARS-CoV-2 infection during the omicron wave in Hong Kong: an observational study. Lancet.

[26] R Core Team (2021). R: a language and environment for statistical computing.

[27] Eggert LE, He Z, Collins W (2022). Asthma phenotypes, associated comorbidities, and long-term symptoms in COVID-19. Allergy.

[28] Chhapola SS (2021). ACE2 expression in allergic airway disease may decrease the risk and severity of COVID-19. Eur Arch Otorhinolaryngol.

[29] Jackson DJ, Busse WW, Bacharier LB (2020). Association of respiratory allergy, asthma, and expression of the SARS-CoV-2 receptor ACE2. J Allergy Clin Immunol..

[30] Morrison CB, Edwards CE, Shaffer KM (2022). SARS-CoV-2 infection of airway cells causes intense viral and cell shedding, two spreading mechanisms affected by IL-13. Proc Natl Acad Sci.

[31] Farne H, Singanayagam A (2020). Why asthma might surprisingly protect against poor outcomes in COVID-19. Eur Respir J..

[32] Takahashi T, Ellingson MK, Wong P (2020). Sex differences in immune responses that underlie COVID-19 disease outcomes. Nature.

[33] Benfante A, Scichilone N (2016). The geriatric asthma: pitfalls and challenges. Asthma research and practice.

[34] Radzikowska U, Ding M, Tan G (2020). Distribution of ACE2, CD147, CD26, and other SARS-CoV-2 associated molecules in tissues and immune cells in health and in asthma, COPD, obesity, hypertension, and COVID-19 risk factors. Allergy.

[35] Nyberg T, Ferguson NM, Nash SG (2022). Comparative analysis of the risks of hospitalisation and death associated with SARS-CoV-2 omicron (B.1.1.529) and delta (B.1.617.2) variants in England: a cohort study. Lancet.

