# Supplementary material for: Association between asthma and COVID-19 severity during Omicron epidemic: a retrospective cohort study using real-world data
Source: BMC Infect Dis. 2024 Jul 4;24:667. doi: 10.1186/s12879-024-09520-9 (PMC11223378; doi:10.1186/s12879-024-09520-9)
Supplement: Supplementary file 1 — Supplementary Material 1. [file 12879_2024_9520_MOESM1_ESM.docx]

**Supplemental Appendix**

**Association between asthma and COVID-19 severity during Omicron epidemic: a retrospective cohort study using real-world data**

1. **Supplemental Figures**

**
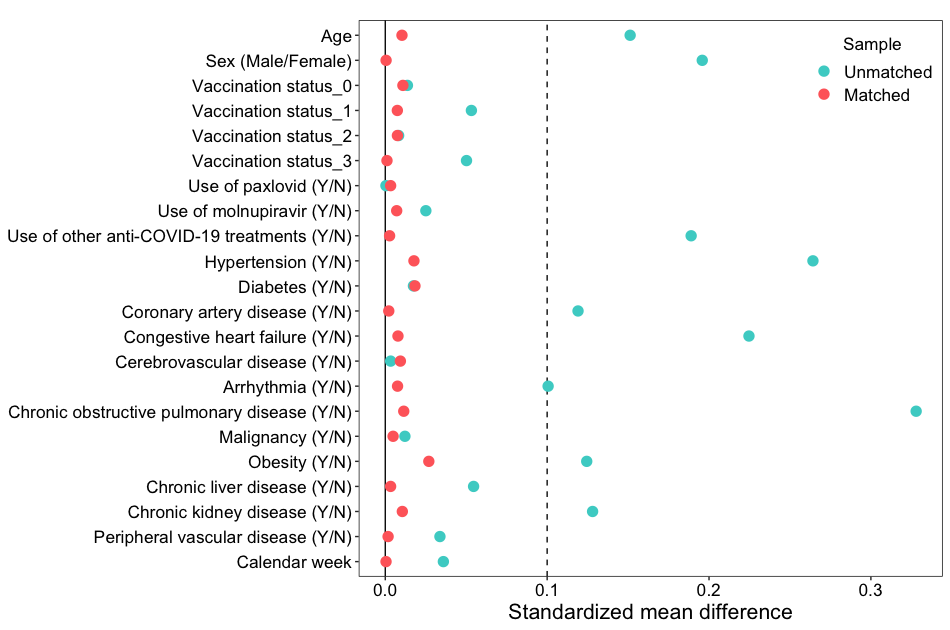
**

**eFigure 1. Standardized mean difference of covariates between COVID-19 patients with and without asthma before and after matching**


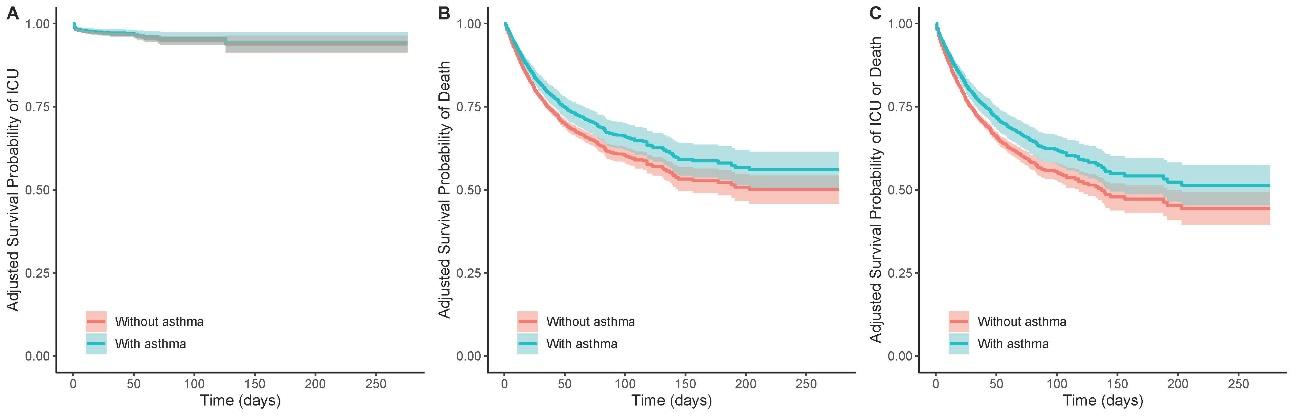


**eFigure 2. Survival distribution of time from the first positive PCR results to the onset of severe clinical outcomes for patients with and without asthma**

The survival probability was determined by using the conditional Cox proportional hazard model adjusted with the covariates. The shaded areas represent 95% confidence intervals.

**
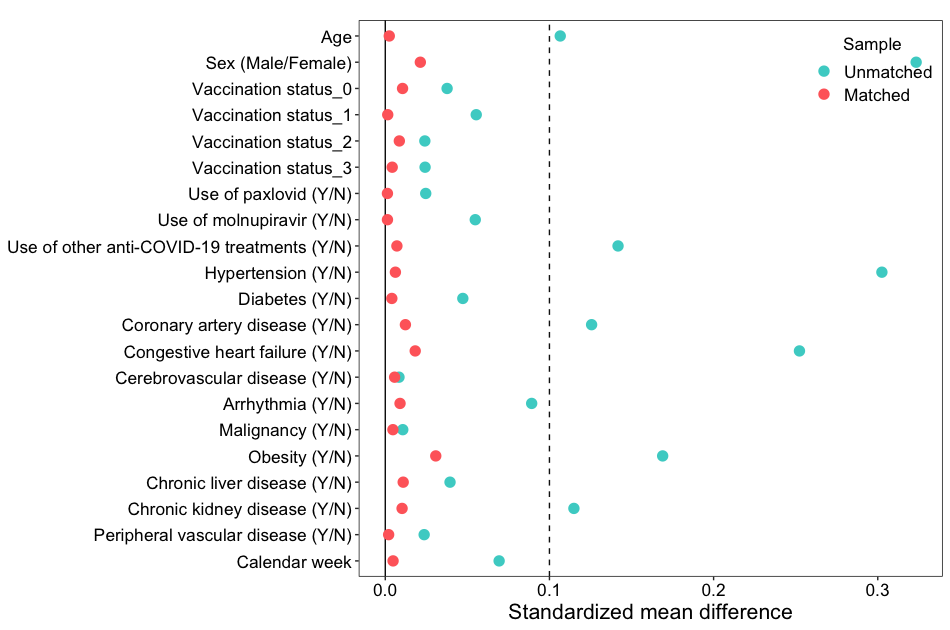
**

**eFigure 3. Standardized mean difference of covariates between COVID-19 patients with and without asthma before and after matching (excluding COPD patients)**

**
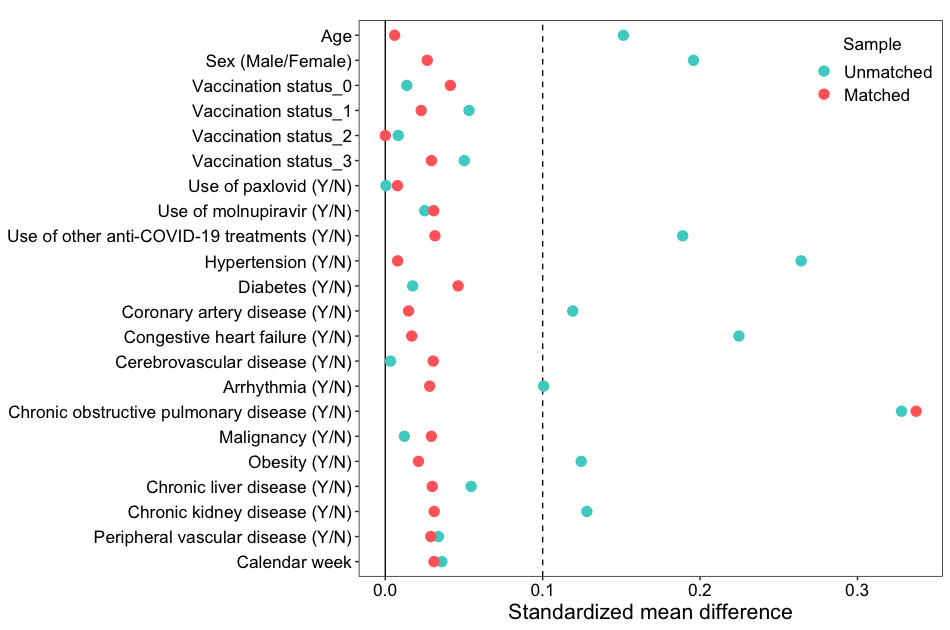
**

**eFigure 4. Standardized mean difference of covariates between COVID-19 patients with and without asthma before and after matching (with matching ratio set at 1:1)**

**
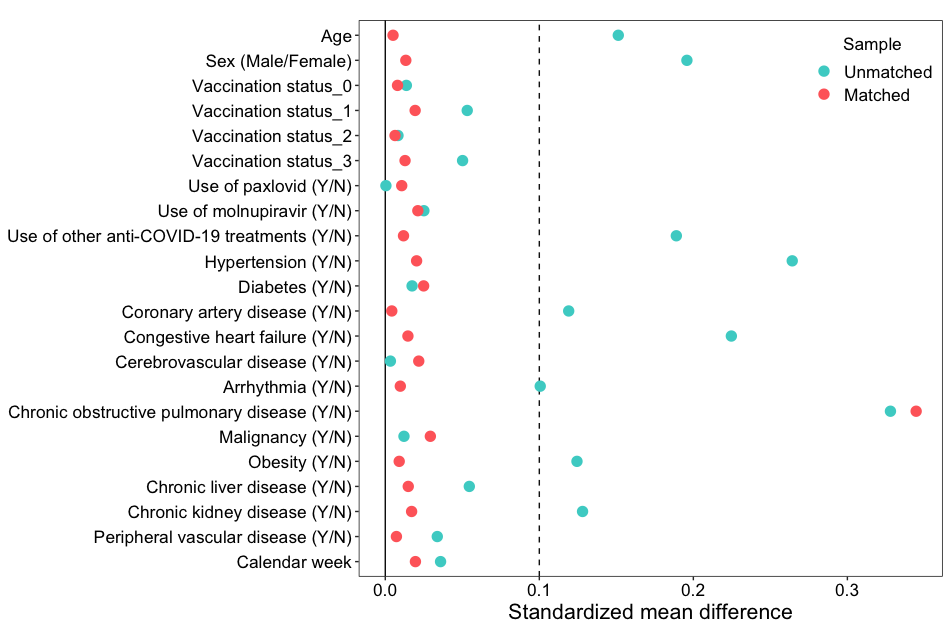
**

**eFigure 5. Standardized mean difference of covariates between COVID-19 patients with and without asthma before and after matching (with matching ratio set at 1:2)**

1. **Supplemental Tables**

**eTable 1. Association between asthma and severe COVID-19 outcomes stratified by asthma therapy**

|  | Without asthma  n (%) | With asthma  n (%) | Crude HR (95% CI) | *p*-value | Adjusted HR (95% CI) ^a^ | *p*-value |
| --- | --- | --- | --- | --- | --- | --- |
| **Patients with asthma and no therapy (n = 57) vs. patients without asthma (n =** **854)** | | | | | | |
| ICU | 29 (3·4) | 1 (1·8) | 0·500 (0·066, 3·804) | 0·503 | 0·466 (0·058, 3·736) | 0·472 |
| Death | 120 (14·1) | 1 (1·8) | 0·115 (0·016, 0·830) | **0·032** | 0·237 (0·031, 1·834) | 0·168 |
| ICU or death | 142 (16·6) | 2 (3·5) | 0·189 (0·046, 0·777) | **0·021** | 0·375 (0·091, 1·546) | 0·175 |
| **Patients with asthma and prescribed ICS + LABA/SABA (n = 509) vs. patients without asthma (n = 7,491)** | | | | | | |
| ICU | 180 (2·4) | 9 (1·8) | 0·734 (0·376, 1·434) | 0·366 | 0·793 (0·406, 1·549) | 0·497 |
| Death | 1067 (14·2) | 57 (11·2) | 0·781 (0·603, 1·010) | **0·060** | 0·799 (0·619, 1·031) | 0·085 |
| ICU or death | 1216 (16·2) | 63 (12·4) | 0·746 (0·583, 0·954) | **0·020** | 0·783 (0·615, 0·998) | **0·048** |
| **Patients with asthma and prescribed ICS + LABA/SABA + LAMA/LTRA/Xanthines (n = 452)**  **vs. patients without asthma (n = 6,268)** | | | | | | |
| ICU | 152 (2·4) | 11 (2·4) | 1·022 (0·556, 1·879) | 0·943 | 1·019 (0·555, 1·869) | 0·952 |
| Death | 1056 (16·8) | 73 (16·2) | 0·926 (0·729, 1·176) | 0·528 | 0·819 (0·641, 1·046) | 0·110 |
| ICU or death | 1174 (18·7) | 81 (17·9) | 0·931 (0·740, 1·171) | 0·542 | 0·837 (0·663, 1·056) | 0·133 |

HR: hazard ratio; CI: confidence interval; ICS: inhaled corticosteroids; LABA: long-acting β2-agonists; SABA: short-acting β2-agonists; LAMA: long-acting muscarinic antagonists; LTRA: leukotriene receptor antagonists

**eTable 2. Association between asthma and severe COVID-19 outcomes after excluding patients with chronic obstructive pulmonary disease**

|  | Without asthma  n = 15,871; n (%) | With asthma  n = 1,081; n (%) | Crude HR (95% CI) | *p*-value | Adjusted HR (95% CI) ^a^ | *p*-value |
| --- | --- | --- | --- | --- | --- | --- |
| ICU | 394 (2.5) | 26 (2.4) | 0.971 (0.652, 1.446) | 0.883 | 0.988 (0.664, 1.469) | 0.952 |
| Death | 2240 (14.1) | 117 (10.8) | 0.755 (0.627, 0.909) | **0.003** | 0.742 (0.616, 0.894) | **0.002** |
| ICU or death | 2561 (16.1) | 133 (12.3) | 0.743 (0.624, 0.885) | **<0.001** | 0.746 (0.626, 0.888) | **<0.001** |

^a^ Adjusted for age, sex, vaccination status, use of paxlovid, molnupiravir and other anti-COVID-19 treatments, medical history, and calendar week

HR: hazard ratio; CI: confidence interval

**eTable 3. Association between asthma and severe COVID-19 outcomes with matching ratio set at 1:1 and 1:2**

|  | Without asthma  n (%) | With asthma  n (%) | Crude HR (95% CI) | *p*-value | Adjusted HR (95% CI) ^a^ | *p*-value |
| --- | --- | --- | --- | --- | --- | --- |
| Matching ratio = 1:1 asthma (n = 1,290) vs. no asthma (n = 1,290) | | | | | | |
| ICU | 42 (3.3) | 28 (2.2) | 0.669 (0.416, 1.078) | 0.099 | 0.691 (0.431, 1.109) | 0.126 |
| Death | 219 (17.0) | 158 (12.2) | 0.728 (0.596, 0.889) | **0.002** | 0.728 (0.593, 0.895) | **0.003** |
| ICU or death | 255 (19.8) | 176 (13.6) | 0.676 (0.560, 0.816) | **<0.001** | 0.690 (0.570, 0.835) | **<0.001** |
| Matching ratio = 1:2 asthma (n = 1,290) vs. no asthma (n = 2,578) | | | | | | |
| ICU | 67 (2.6) | 28 (2.2) | 0.835 (0.537, 1.299) | 0.424 | 0.821 (0.530, 1.273) | 0.379 |
| Death | 416 (16.1) | 158 (12.2) | 0.760 (0.637, 0.907) | **0.002** | 0.758 (0.631, 0.910) | **0.003** |
| ICU or death | 469 (18.2) | 176 (13.6) | 0.735 (0.622, 0.869) | **<0.001** | 0.738 (0.622, 0.876) | **<0.001** |

^a^ Adjusted for age, sex, vaccination status, use of paxlovid, molnupiravir and other anti-COVID-19 treatments, medical history, and calendar week

HR: hazard ratio; CI: confidence interval

**eTable 4. Association between asthma and severe COVID-19 outcomes stratified by the most recent ICS dose**

|  | Without asthma  n (%) | With asthma  n (%) | Crude HR (95% CI) | *p*-value | Adjusted HR (95% CI) ^a^ | *p*-value |
| --- | --- | --- | --- | --- | --- | --- |
| **Patients with asthma not prescribed ICS (n = 339) vs. patients without asthma (n = 5,026)** | | | | | | |
| ICU | 121 (2.4) | 9 (2.7) | 1.095 (0.553, 2.168) | 0.794 | 1.047 (0.528, 2.077) | 0.895 |
| Death | 743 (14.8) | 29 (8.6) | 0.572 (0.397, 0.824) | **0.003** | 0.669 (0.459, 0.977) | **0.037** |
| ICU or death | 842 (16.8) | 34 (10.0) | 0.580 (0.415, 0.812) | **0.002** | 0.675 (0.480, 0.950) | **0.024** |
| **Patients with asthma prescribed low-dose ICS (n = 295) vs. patients without asthma (n = 4,289)** | | | | | | |
| ICU | 101 (2.4) | 3 (1.0) | 0.457 (0.143, 1.459) | 0.186 | 0.498 (0.155, 1.600) | 0.242 |
| Death | 664 (15.5) | 34 (11.5) | 0.823 (0.582, 1.162) | 0.268 | 0.869 (0.615, 1.229) | 0.428 |
| ICU or death | 741 (17.3) | 35 (11.9) | 0.732 (0.522, 1.028) | 0.072 | 0.783 (0.562, 1.092) | 0.150 |
| **Patients with asthma prescribed medium-dose ICS (n = 488) vs. patients without asthma (n = 6,918)** | | | | | | |
| ICU | 170 (2.5) | 10 (2.0) | 0.812 (0.429, 1.540) | 0.524 | 0.826 (0.439, 1.555) | 0.554 |
| Death | 1078 (15.6) | 74 (15.2) | 0.874 (0.698, 1.096) | 0.244 | 0.783 (0.621, 0.987) | **0.038** |
| ICU or death | 1216 (17.6) | 80 (16.4) | 0.838 (0.674, 1.044) | 0.115 | 0.770 (0.616, 0.963) | **0.022** |
| **Patients with asthma prescribed high-dose ICS (n = 168) vs. patients without asthma (n = 2,408)** | | | | | | |
| ICU | 62 (2.6) | 6 (3.6) | 1.455 (0.652, 3.245) | 0.360 | 1.465 (0.639, 3.357) | 0.367 |
| Death | 362 (15.0) | 21 (12.5) | 0.836 (0.531, 1.318) | 0.441 | 0.805 (0.509, 1.273) | 0.353 |
| ICU or death | 414 (17.2) | 27 (16.1) | 0.962 (0.638, 1.452) | 0.854 | 0.937 (0.623, 1.410) | 0.756 |

^a^ Adjusted for age, sex, vaccination status, use of paxlovid, molnupiravir and other anti-COVID-19 treatments, medical history, and calendar week

ICS: inhaled corticosteroids; HR: hazard ratio; CI: confidence interval
